# The Surgical Specialty1President’s address delivered before the 35th annual meeting of the Medical Association of Central New York, held at Syracuse, N.Y., October 21, 1902.

**Published:** 1902-12

**Authors:** A. L. Beahan

**Affiliations:** Canandaigua, N.Y.


**BUFFALO MEDICAL JOURNAL.**

VOL. XLII.—LVIII. DECEMBER, 1902. No. 5.

### ORIGINAL COMMUNICATIONS.

### The Surgical Specialty.[1]

By A. L. BEAHAN, M.D., Canandaigua, N.Y.

SINCE Lister promulgated his doctrine of antiseptic treatment of wounds, surgery has been revolutionised. In the past thirty-seven years his teachings have been followed with such success that surgery as a specialty has been placed upon a solid basis, and the truths then enunciated have become classical. The principle of thorough cleanliness, with the prevention of wound contamination by proper antiseptics and exclusion of particles from the air that prevent by their development and consequent healing of wounds and restoration to normal conditions of the cavities of the body, has been brought forward to a scientific position, precise and unassailable.

The work of Tait in abdominal surgery gave a technique of matchless character and results that astonished the world. The principles evolved taught us much of new value in all lines of surgical success. His was the master mind. He not only marked out new paths with originality and force, but of the varied procedures and from different methods he sifted out the best plans and adopted the safest and most successful courses to pursue. If the one fallacy, the opium treatment of peritonitis, the Meigs method, had he only and alone exploded, his name were written high up on the monument of the world's benefactors. But in the matter of drainage of wounds, a principle of Lister's and founded on the necessity for preventing accumulations and elaboration of morbid products, Tait established and popularised a principle that stands the severe test of experience

1. President's address delivered before the 35th annual meeting of the Medical Association of Central New York, held at Syracuse, N.Y., October 21, 1902.

and after much wandering of recent pathological thought, a gradual strengthening of this truth is felt.

It is a comfort to feel that confidence in unchanging principles is unshaken; that a difference in technique is a difference of detail only. Cleanliness and simplicity should be sought for first, last and always in the province of surgery. To invent a new instrument is not so important as to make an old one do more work. The man who cannot be a surgeon with a jack-knife can never be a surgeon. The surgical specialty as a topic should deal more with men and their work in this department of medicine rather than measures and principles. It may be fairly stated that surgical work is being widely disseminated. It is not controlled by colleges, nor by the hospital staffs. After a stress period of ovarian operations came appendiceal, next kidney and gall-bladder surgery claimed attention.

The old dictum that a long service in medicine should precede the choosing of any specialty, was in part wise, but it does not hold as true with a four years' course of study as it did with two courses of five months each. Surgical work is now in the hands of the many and it will so continue. It is safest when so adjusted. The education of the people on true scientific surgical lines is growing. Their appreciation of fine discrimination is acute. Who has not had children make the diagnosis of appendicitis in their own cases? What physician dare say to his patrons, "Your kinsman or friend died of peritonitis," without making an explanation of its cause? No doubt the rapid advancement of surgical knowledge and the fascination of its successful features in attracting so large a number of operators may have given the aggregate of individual records an increase of failures, compared to what successes might be recorded if the greater operators could have corralled and controlled the whole of the clinical material. But excesses always correct themselves and the intensities and vital forces that with strenuous energy work out new problems can be trusted to finally subside to normal limits.

It is growing to be a question, if, on surgical lines, one can afford to exploit a moderate talent in a limited field. Surgery is often the final spasm of effort in a forlorn hope and it weighs on the consciences of pursuers of the healing art and provokes fierce criticisms by the laity when failure occurs. But when the operators of any hospital for any month nearly equal the number of operations, it is a question whether dissemination of surgical science has not reached its limit. The effect of this democratic plan of rendering available for all aspirants to surgical honor or emolument, the hospital service and equipment has features worth further studying. If infection occurs and spreads, to trace its source, if from without the institution, becomes impossible. Because of general permission without supervision or responsibility, incompetency is not excluded, nor controlled. The private patient suffers most, the ward patient least, because the latter must be served by one of the staff. But no one of the several attending surgeons gains sufficient patronage to develop a clinic of such importance that the education of the profession is advanced.

The pushing, pressing, over-crowded profession of medicine and surgery develops in the individual member the desire for fame and a procedure or an instrument is labeled with the name of its user or inventor and "my" or "mine" becomes greater than the greatest. Simple technique is abandoned for complex, common plans for complicated, the new way for the old. Of instruments there is no end. What may be needed is asked on planning for an operation, not what is required, what indispensable. Take the needle alone as an illustration. The scythe-like Hagadorn, cutting a swath of cradle-like proportions. The Peaslee, a threaded push and pulling lacerating grotesque instrument. The Glover, where the point is larger than the body and the cutting edges extend half way back to the hilt, causing a puncture and incision that altogether would carry several times as much thread as the needle holds. Each of these or their similars, as given us by the manufacturers, should be discarded either because of their uselessness or bad construction, or because they are needless profusions of our armamentarium. The history of surgery shows repetitions of principles, but few new discoveries. Each operation exhausts itself, giving way to some other popular work. Careful observation is needed to not follow will o' the wisps in surgery.

It is doubtful if Emmet's trachelorrhaphies and perineorrhaphies, Bantock's suprapubic serreneud extraperitoneal amputations and stump treatments of uterine fibroids; Buck's extension for fracture of the femur; Moore's method in Colles's fracture, reduction and treatment; Mayo Robson's technique in gall-bladder operations will ever be successfully improved.

It is not very wise to rush after confusing and diverting and really unimportant changes in technique. "I have an operation of my own" is an exclamation tiresome, vain and contains a false note that grates the ear or palls the taste. "Medicine is a science, surgery a divine art," exclaimed a reader at the American Medical Association, at the Denver meeting. "Jesus Christ and I cure," said Dr. Dick Levis, years since in a public address. What magnifying of human powers, what blasphemy! True conservatism does not hesitate, but is never bold; does not assume to possess unwarranted ability nor scope, but in humble, patient, conscientious labor works out true scientific results; does not play to the galleries, but points out with honest thought the hard and fast principles of the thorny road to successful achievement or possible, but honorable, defeat. Dr. McMurtry at a section dinner at Atlantic City, at a meeting of the American Medical Association, stated that he sincerely pitied the abdominal surgeon in his first hundred cases; that no other work in the world was so intense. The door bell at night caused the cold perspiration to start over the whole body. The nerve tension was frightful.

The surgical specialty comprises no smooth road, conduces to no peace of mind. No drone, no dullard can succeed in it, nor should. The warp, the woof, the finished product must absorb the whole of the mentality, be the consecration of the complete, full-fledged faculties. The finger tips need supersensitiveness; the mind must not be diverted; a medical man, the husband of a patient on whom Tait was operating for ovarian disease asked Tait a question during the course of the operation. The knife went wrong and the patient nearly lost her life from the hemorrhage of a cut artery.

The gratifications of success, not the mercenary end must be the payment for much of service. Surgery is a necessity, not as medicine, a choice. It must face consequences, as of maiming for life, or causing untold suffering. The die is surely cast when the operation is completed. It is either weal or woe. There can be no checking, no hedging. The responsibility has no parallel, because of its intensity, in the purely medical branch of service. Self-limitation does not enter into the calculation when the knife cures. Principles must not be broken. The steady hand, the clear brain, the correct eye, the balanced judgment, trained for wise comprehension and acute action must be alert and ready. The work of the moment transcends in importance everything else in the world and absorbs every thought and emotion of being. The achievements of American surgeons and the status of the surgical specialty in our own country are very important. Anglo-Saxon courage, resource and ability have written many new chapters in surgical progress. Genius, invention, quick application and clearness of discrimination may be claimed as our characteristics. The universality of education, the wide-spread distribution of journals and books, the establishment and strong support of societies for fostering research, comparison of work, mutual benefit and social intercourse, make American medicine in all its branches progressive and of virile power.

The history of this society demonstrates the increased usefulness of such organisations in its increased membership and interest in meetings. Since we last met the first president of this society, in 1868, Dr. Edward Mott Moore, of Rochester, has died. He was at once the dean and patriarch of medicine in the section and boundaries represented here. In original research, love of his profession, its literature, and practice, helpfulness to fellow-workers, breadth of character, purity of life, tenacity of purpose, Dr. Moore's record stands high. His reputation became world-wide when the first correct interpretation and treatment of Colles's fracture was by him properly made.

It is recommended that suitable resolutions be presented at this time, commemorative and expressive of the regard of this society for Dr. Moore.

A new feature has been added to this present meeting, a banquet. It is the belief of your officers that this feature, by merging social with literary elements, will strengthen the society.

Thanking you for the honor of being permitted to preside over your deliberations, it is my pleasure to declare this, the thirty-fifth annual meeting of the Medical Association of Central New York, open for such business as you its members may transact and to invite your attention to the program offered.

